# 脱水（干瘪）遗传性口型红细胞增多症23例：2018–2024年北京协和医院单中心回顾性队列研究

**DOI:** 10.3760/cma.j.cn121090-20250312-00132

**Published:** 2025-11

**Authors:** 琦 王, 昊 蔡, 丽灵 林, 剑 李, 冰 韩, 苗 陈

**Affiliations:** 1 中国医学科学院、北京协和医学院、北京协和医院血液内科，北京 100730 Department of Haematology, Peking Union Medical College Hospital, Chinese Academy of Medical Sciences and Peking Union Medical College, Beijing 100730, China; 2 中国医学科学院、北京协和医学院、北京协和医院检验科，北京 100730 Department of Clinical Laboratory, Peking Union Medical College Hospital, Chinese Academy of Medical Sciences and Peking Union Medical College, Beijing 100730, China

**Keywords:** 遗传性口型红细胞增多症, 基因型, 铁过载, Dehydrated hereditary stomatocytosis, Genotype, Iron overload

## Abstract

**目的:**

由PIEZO1或KCNN4基因突变引起的脱水（干瘪）遗传性口型红细胞增多症（Dehydrated hereditary stomatocytosis, DHS）罕见，本研究回顾性分析DHS队列临床特征、诊断、基因型及铁过载发生情况，以提高DHS诊治水平。

**方法:**

纳入了2018–2024年在北京协和医院因疑诊先天性溶血性贫血行基因二代测序发现PIEZO1或KCNN4基因致病突变并诊断为DHS的患者，收集并分析患者临床特征、基因型、铁过载并发症及治疗结局。

**结果:**

共纳入23例DHS患者，其中PIEZO1突变21例，KCNN4突变2例。临床表现为脾大、黄疸、胆石症和继发铁过载，8.7％的患者出现轻度贫血，17.4％的患者出现中度贫血。仅17.4％的患者血涂片中可见数量不等的口型红细胞。从出现症状至确诊中位时间11年。PIEZO1基因突变以c.7367G>A、c.6328C>T为主，发现9个首次报道的突变位点。KCNN4基因均为c.1055G>A基因型。患者基因型与表型无明确相关性。21.7％的患者继发铁过载，肝脏为主要受累脏器。

**结论:**

DHS容易漏诊、误诊，基因检测有助于快速确诊。DHS随访过程中应监测继发性肝脏铁过载。

## Abstract (en)

**Objective:**

Dehydrated hereditary stomatocytosis（DHS）, a rare condition caused by pathogenic PIEZO1 or KCNN4 mutations, remains under-recognized. This study aimed to improve the diagnosis and management of DHS in China by retrospectively analyzing clinical and genetic characteristics.

**Methods:**

Patients diagnosed with DHS at Peking Union Medical College Hospital between 2018 and 2024 were included. All had suspected congenital hemolytic anemia and were confirmed by next-generation sequencing to harbor pathogenic PIEZO1 or KCNN4 mutations. Clinical features, genotypes, iron overload, and treatment outcomes were collected and analyzed.

**Results:**

Twenty-three DHS patients were identified, including 21 with PIEZO1 and 2 with KCNN4 mutations. Common manifestations were splenomegaly, jaundice, cholelithiasis, and secondary iron overload. Mild and moderate anemia occurred in 8.7％ and 17.4％ of patients, respectively, and stomatocytes were present in only 17.4％. The median time from symptom onset to diagnosis was 11 years. The predominant PIEZO1 variants were c.7367G > A and c.6328C > T, with nine novel mutations identified. Both KCNN4 mutations involved c.1055G > A. No clear genotype–phenotype correlation was observed. Secondary hepatic iron overload was found in 21.7％ of patients.

**Conclusion:**

DHS is often underdiagnosed or misdiagnosed. Genetic testing enables accurate identification, and regular monitoring for secondary hepatic iron overload is crucial for long-term management.

遗传性口型红细胞增多症（Hereditary stomatocytosis, HST）是由于红细胞膜对阳离子通透性增加而导致红细胞体积失调、形态异常的一组先天性溶血性疾病[1]。在因部分患者外周血涂片中可以看到数量不等的口型红细胞而得名。2018年Andolfo等[1]将HST分为非综合征型（孤立性红系表型）和综合征型（血液学外表现），非综合征型包括过度水化（水肿）型HST、冰冻红细胞增多症、脱水（干瘪）遗传性口型红细胞增多症（Dehydrated hereditary stomatocytosis, DHS）以及家族性假性高钾血症。综合征型包括Stomatin缺陷的冷水细胞增多症伴智力障碍、癫痫发作和肝脾肿大；植物甾醇血症裂红细胞症与巨血小板减少症；DHS伴围生期水肿和（或）假性高钾血症[1]。DHS是HST中最常见的类型，发病率约为1∶50 000[2]，为常染色体显性遗传病，多数患者表现为代偿性溶血和脾大，容易继发铁过载。

PIEZO1或KCNN4基因突变使红细胞膜对一价阳离子Na^+^和K^+^的通透性改变，导致细胞脱水、形成口型红细胞，分别引起DHS 1型和DHS 2型[3]–[5]。DHS 1型和DHS 2型临床表现相似，但严重表型各不相同，DHS 1型患者更易出现铁过载，在接受脾切除术后更易发生血栓事件。而DHS 2型患者HGB更低、黄疸更重[1]。

因为DHS的罕见性，目前仅意大利2018年报道了一个较大的HST队列，其中29个家庭为PIEZO1或KCNN4基因突变的DHS[6]。在亚洲地区，日本曾描述了一组12个家庭DHS的PIEZO1或KCNN4基因突变[7]；中国尚无DHS发病率数据，2017年邵英起等[8]首次报道了主要通过外周血涂片存在5％以上口型红细胞诊断的12例HST临床特征，未行基因检测故未判定具体类型；2019年李园等[9]报道了5例PIEZO1基因突变的DHS患者特征；两组患者均表现为溶血伴黄疸、脾大，无患者继发铁过载。本研究纳入了23个无关家庭的PIEZO1或KCNN4基因突变的DHS患者，对DHS临床特点、诊断、基因型及铁过载并发症分析如下。

## 病例与方法

一、病例资料

本研究为单中心回顾性队列研究，纳入了2018年至2024年在北京协和医院因疑诊先天性溶血性贫血行基因二代测序检查，发现PIEZO1或KCNN4基因致病突变并诊断为DHS的患者。渗透压梯度埃克塔细胞测量术（暂定名）是诊断遗传性红细胞膜通透性障碍性疾病的关键试验[2],[10]，鉴于中国无法进行上述检验，本研究定义DHS的诊断依据为：①临床表现为不同程度的黄疸、脾脏肿大，伴或不伴贫血；②实验室检查符合溶血性黄疸，外周血涂片有或没有口型红细胞；③基因测序存在PIEZO1或KCNN4基因致病突变；④排除其他先天性和获得性溶血性贫血。使用QIAamp DNA血液分离试剂盒从外周血中分离的基因组DNA进行二代测序，以鉴定致病性PIEZO1基因突变、KCNN4基因的功能获得性突变以及与其他DHS类型相关的突变、其他先天性溶血性贫血及先天性血色病基因突变。通过家族史、红细胞膜及酶病相关检测、抗人球蛋白试验等协助排除其他溶血疾病。

二、数据采集

收集人口统计信息，包括性别、年龄、出生地和病史（症状、发病时间、诊断方法和家族史）。收集的实验室检测结果包括全血细胞计数、平均红细胞体积（MCV）、平均红细胞血红蛋白量（MCH）、平均红细胞血红蛋白浓度（MCHC）、外周血涂片、乳酸脱氢酶（LDH）、总胆红素（TBIL）、间接胆红素（IBIL）、血浆游离血红蛋白、红细胞锌原卟啉、铁蛋白（SF）、血清铁（SI）、转铁蛋白饱和度（TS）、总铁结合力（TIBC）、叶酸、维生素B_12_、红细胞渗透脆性、6磷酸葡萄糖脱氢酶（G-6-PD）、血红蛋白电泳、酸溶血及Ham试验、Coombs试验、先天性溶血性疾病及血色病基因二代测序报告。影像学检查包括腹部超声、脏器（肝脏、心脏、脾脏、胰腺）T2加权铁负荷定量核磁共振（T2* MRI）。

根据WHO指南[11]，成人贫血严重程度按HGB水平分为3级，轻度贫血：HGB 110～119 g/L（女性）或110～129 g/L（男性）；中度贫血：HGB 80～109 g/L；重度贫血：HGB<80 g/L。溶血严重程度按血清总胆红素（TBIL）水平分为3级，轻度溶血：TBIL>17.10 µmol/L且≤42.75 µmol/L；中度溶血：TBIL>42.75 µmol/L且≤85.50 µmol/L；重度溶血：TBIL>85.50 µmol/L。肝功能损伤程度按丙氨酸转氨酶（ALT）与天冬氨酸转氨酶（AST）中较高者分为3级，轻度肝功能损伤：ALT或AST>40 U/L且≤120 U/L；中度肝功能损伤：ALT或AST>120 U/L且≤200 U/L；重度肝功能损伤：ALT或AST>200 U/L。

红细胞渗透脆性试验结果正常参考范围分别为：开始溶血：0.45％～0.50％ NaCl溶液（部分红细胞膜破裂，上清液呈淡红色）；完全溶血0.30％～0.35％ NaCl溶液（>90％红细胞溶解，溶液呈透明红色）。当溶血浓度>正常上限，则提示红细胞渗透脆性增高，当溶血浓度<正常下限，则提示红细胞渗透脆性减低。DHS患者红细胞因膜蛋白缺陷导致细胞内K^+^外流增加，细胞脱水、体积缩小，表面积/体积比降低，细胞膜张力减小，在低渗盐水中表现出抵抗力增强，溶血盐浓度降低，渗透脆性降低。即与正常红细胞相比，口型红细胞在更低渗的溶液中才发生破裂[11]。

三、铁过载评估

在排除活动性炎症、肿瘤和酗酒等因素的影响后，SF>1 000 µg/L、TS>45％（男性）或50％（女性）诊断为铁过载[13]，合并肝病患者，还需有肝穿刺活检病理或T2* MRI证实存在肝脏铁负荷增多[14]。本组铁过载患者均行MRI测定脏器（肝脏、心脏、脾脏、胰腺）T2*值定量评估各脏器铁负荷，分级标准如下：肝脏铁过载：T2*值>6.3 ms为正常，1.4～6.3 ms为轻度，<1.4 ms为重度；心脏铁过载：T2*值>20 ms为正常，10～20 ms为轻度，<10 ms为重度。T2*值越低，铁负荷越高。

四、统计学处理

使用频率和百分比描述分类数据。正态分布的连续变量表示为平均值和标准差，而非正态分布变量表示为中位数和四分位数范围（*IQR*）。SPSS 27.0用于统计分析，采用Wilcoxon符号秩和检验比较两独立样本组间差异，双侧*P*<0.05为差异有统计学意义。

## 结果

一、患者一般特征

本研究共纳入23例DHS患者，其中男性13例（56.5％）、女性10例（43.5％）；DHS 1型21例、DHS 2型2例。患者确诊的中位年龄31.0（*IQR* 19.0～40.0）岁。从出现症状到确诊的中位病程11.0（*IQR* 4.0～20.5）年。患者多因黄疸、脾大就诊，主要临床表现为：皮肤巩膜黄染（22/23，95.7％），脾大（18/23，78.3％）、胆石症（10/23，43.5％）、贫血（6/23，26.1％）、铁过载（5/23，21.7％）。2例患者曾行脾切除术，1例患者曾行脾部分栓塞术。7例患者曾行胆囊切除术。23例患者中4例（17％）家族史信息无法获得，余19例患者中7例明确存在阳性家族史，12例否认家族史（其中2例患者行家系基因测序提示父母无相关基因突变，患者为新发突变；另10例患者未行家系基因测序）。无患者父母为近亲婚配。

二、实验室检查

23例患者白细胞及血小板水平均正常。中位HGB水平为132.5（*IQR* 111.2～151.5）g/L，6例（26.1％）患者出现贫血，其中2例为轻度贫血，4例为中度贫血，其余17例（73.9％）表现为代偿性溶血。全组患者中位MCV为92.1（*IQR* 87.6～105.6）fl，中位MCH为34.8（*IQR* 31.3～37.6）pg，中位MCHC为374.0（*IQR* 370.3～377.8）g/L，贫血类型为正细胞性。所有患者均存在溶血，4例（17.4％）为轻度溶血，12例（52.2％）为中度溶血，7例（30.4％）为重度溶血。中位网织红细胞（Ret）百分比6.07％（*IQR* 3.92％～10.70％），中位LDH 197.5（*IQR* 170.3～263.0）U/L，中位TBIL 68.9（*IQR* 56.9～101.0）µmol/L，中位IBIL 59.0（*IQR* 48.9～91.0）µmol/L。

仅4例（17.4％）患者的外周血涂片中可见口型红细胞，其中1例口型红细胞占30％，3例口型红细胞占1％～5％。3例（13.0％）可见球形红细胞，2例（8.7％）可见Pappenheimer小体，1例（4.3％）可见干瘪红细胞呈靶形改变。14例（60.9％）外周血涂片大致正常，未看到异常形态的红细胞。20例患者行红细胞渗透脆性试验，14例患者溶血盐浓度正常，1例患者溶血盐浓度降低，5例患者溶血盐浓度升高。9例患者进行了血浆游离血红蛋白的检测，其中4例水平升高。其他溶血试验包括G-6-PD活性、血红蛋白电泳相关检测均在正常范围内。

三、基因突变分析

二代测序发现PIEZO1等位基因突变21例，KCNN4等位基因突变2例。其中20例（87.0％）为错义突变，3例（13.0％）为重复/缺失。PIEZO1基因突变患者中5例为PIEZO1 c.7367G>A基因型，2例患者为PIEZO1 c.6328C>T基因型，其余突变位点各见于1例患者，基因型各不相同。本队列中有9个PIEZO1基因位点新突变为首次报道，包括c.1815G>T、c.4072C>T、c.2492C>T、c.3796+7G>A、c.6067C>G、c.5482G>C、c.553del、c.7483_7488dupCTGGAG、c.1668del。2例KCNN4基因突变患者均为c.1055G>A基因型（表1）。

**表1:** 23例脱水（干瘪）遗传性口型红细胞增多症患者致病基因分析

例号	性别	年龄（岁）	致病基因	突变位点及蛋白质
1	女	54	PIEZO1	c.1668del（p.Glu557Serfs*16）
2	女	40	PIEZO1	c.1815G>T（p.Met605Lle）
3	女	17	PIEZO1	c.2492C>T（p.Ser831Leu）
4	男	16	PIEZO1	c.3796+7G>A（splicing）
5	女	40	PIEZO1	c.4072C>T（p.Arg1358Cys）
6	男	29	PIEZO1	c.5482G>C（p.Glu1828Gln）
7	女	24	PIEZO1	c.553del（p.Ala185Profs*21）
8	男	40	PIEZO1	c.6008C>A（p.Ala2003Asp）
9	女	36	PIEZO1	c.6059C>T（p.Ala2020Val）
10	男	28	PIEZO1	c.6067C>G（p.Leu2023Val）
11	男	19	PIEZO1	c.6380C>T（p.Thr2127Met）
12	男	17	PIEZO1	c.6328C>T（p.Arg2110Trp）
13	男	60	PIEZO1	c.6328C>T（p.Arg2110Trp）
			UGT1A1	c.-41_-40dup（NA）
14	女	35	PIEZO1	c.7150G>A（p.Gly2384Ser）
15	女	31	PIEZO1	c.7367G>A（p.Arg2456His）
16	男	19	PIEZO1	c.7367G>A（p.Arg2456His）
17	男	14	PIEZO1	c.7367G>A（p.Arg2456His）
18	男	42	PIEZO1	c.7367G>A（p.Arg2456His）
19	男	32	PIEZO1	c.7367G>A（p.Arg2456His）
			UGT1A1	c.3263T>G（NA）
20	女	42	PIEZO1	c.7463G>A（p. Arg2488Gln）
21	女	38	PIEZO1	c.7483_7488dupCTGGAG（p.Leu2495_Glu2496dupLeuGlu）
22	男	28	KCNN4	c.1055G>A（p.Arg352His）
23	男	29	KCNN4	c.1055G>A（p.Arg352His）

**注** NA：未进行或未成功检测到相应的蛋白质改变

23例患者均未检测出合并其他先天性溶血性贫血及血色病的致病基因异常。2例患者为PIEZO1基因突变合并UGT1A1致病基因突变，分别为PIEZO1 c.6328C>T合并UGT1A1 c.-41_-40dup、PIEZO1 c.7367G>A合并UGT1A1 c.3263T>G基因型，此2例患者TBIL分别为140.5 µmol/L和80.5 µmol/L，黄疸程度较其他患者（中位TBIL 65.25 µmol/L）明显加重。2020年我们曾报道其中1例DHS合并Gilbert综合征继发铁过载[15]。

四、DHS继发铁过载的特征

23例DHS患者中位SF水平为276.0（*IQR* 89.3～1 085.5）µg/L，中位TS水平为84％（*IQR* 33.9％～91.0％），中位TIBC水平为237.0（*IQR* 216.0～336.0）µg/dl。5例（21.7％）患者继发铁过载，其中4例为DHS 1型，1例为DHS 2型，中位SF水平为1 917.0（*IQR* 1 003.0～2 951.5）µg/L，中位TS水平为91.0％（*IQR* 70.1％～94.1％）。5例患者的心功能（左心室射血分数）、甲状腺功能及糖化血红蛋白均处于正常范围内。铁过载主要受累脏器为肝脏，4例患者出现肝功能损伤，其中1例（25％）为轻度肝功能损伤，3例（75％）为重度肝功能损伤（其中2例继发门静脉高压表现）。T2* MRI定量评估显示4例患者肝脏重度铁沉积（低于软件检测下限），1例患者肝脏轻度铁沉积（肝脏T2*值约2.8 ms）；1例患者心肌受累，为轻度铁过载（心肌室间隔中部取样T2*信号强度为16.9 ms）；胰腺、脾脏T2*值均在正常范围。2例患者因黄疸、肝功能异常原因不明行肝脏穿刺活检，病理提示肝细胞弥漫浊肿，部分气球样变，少量肝细胞点状坏死，肝细胞内可见大量含铁血黄素沉积（普鲁士蓝铁染色+、脂褐素染色-），汇管区淋巴细胞为主炎细胞浸润，纤维组织增生，部分纤维插入肝小叶内（Masson三色染色+、网状纤维染色+），可见窦周纤维化，符合铁过载肝内含铁血黄素沉积表现。DHS继发铁过载患者肝功能损伤发生率明显高于未继发铁过载患者（3/5对0/18，Fisher，*P*＝0.006）。

五、治疗和患者结局

本组贫血患者均为轻度贫血，未针对贫血进行治疗，叶酸缺乏的患者给予补充口服叶酸。黄疸、脾大患者均未采取治疗，不影响日常一般生活。23例患者诊断后中位随访38.0（*IQR* 19.3～48.8）个月，所有患者全部存活。

5例继发性铁过载的患者接受去铁治疗，均口服地拉罗司或去铁酮治疗，其中1例采用静脉放血治疗，中位治疗7.0（*IQR* 6.0～9.5）个月，中位SF下降至445.9（*IQR* 125.5～898.6）µg/L。4例患者在去铁治疗6～12个月后复查T2* MRI，其中3例患者肝脏、心脏T2*值均治疗前上升。2例患者去铁治疗后HGB水平较前上升（表2）。

**表2:** 5例铁过载患者去铁治疗前后铁负荷相关指标变化

序号	突变基因名称及位点	去铁治疗方案	去铁治疗前				去铁治疗后			
			SF（µg/L）	HGB（g/L）	肝脏T2*（ms）	心脏T2*（ms）	SF（µg/L）	HGB（g/L）	肝脏T2*（ms）	心脏T2*（ms）
1	PIEZO1c.6059C>T	地拉罗司	2 463	95	<LOD	24.6	88	104	12.4	27.9
2	PIEZO1c.6008C>A	地拉罗司	1 917	145	1.3	25.1	163	132	–	–
3	PIEZO1c.7367G>A	去铁酮、地拉罗司	1 838	145	2.8	30.0	182	122	3.4	28.8
4	PIEZO1c.7367G>A UGT1A1c.3263T>G	放血；去铁酮、地拉罗司	3 440	97	<LOD	16.9	821	127	<LOD	22.7
5	KCNN4 c.1055G>A	地拉罗司	1 168	124	<LOD	26.8	975	106	0.9	36.2

**注** <LOD：肝脏T2*信号强度明显降低，低于软件检测下限；SF：铁蛋白；T2*：T2加权铁负荷定量

2例PIEZO1突变的DHS 1型患者分别在37岁、40岁行脾切除手术。1例KCNN4突变的DHS2型患者12岁时曾行脾栓塞术，至28岁彩超显示脾脏缩小（厚5.2 cm，长6.9 cm）。3例患者术后血红蛋白水平及胆红素水平均较前改善，3例患者术后均未抗凝治疗，分别随访至术后4、2和18年，均未发生血栓事件。此3例患者术前均因误诊为遗传性球形红细胞增多症（HS）而行手术治疗。

## 讨论

本研究总结了23例中国单中心PIEZO1或KCNN4基因突变所致DHS患者的临床特征，为这一罕见疾病的诊疗提供了参考。

DHS临床表现异质性高，可从无症状到严重溶血[6]，典型病例依赖血涂片中大量口型红细胞进行诊断。无明显贫血，血涂片无典型口型红细胞，甚至可见球形红细胞的情况下，则容易误诊[10],[16]。本组患者仅17.4％可见口型红细胞，多数表现为代偿性溶血，伴黄疸、脾大，贫血发生率仅为26.1％。患者自出现症状至确诊中位延迟达11年，突显其诊断难度。口型红细胞检出率低可能与红细胞脱水程度不一、形态变异（如球形或靶形红细胞）及涂片判读经验有关。红细胞渗透脆性试验在本队列中大多正常，仅1例降低，与既往报道中DHS常表现为渗透脆性下降有所不同[17]。这可能与患者外周血中口型红细胞比例较低有关，其整体渗透抗性被正常红细胞所掩盖[18]。

基因检测是确诊DHS的关键。本组以PIEZO1突变为主，c.7367G>A及c.6328C>T为常见基因型，与欧美报道一致[6]，但在中国属首次报道。DHS 2型仅有十多个病例报道，c.1055G>A基因型占50％以上[19]。2024年中国首例[20]和本队列的2例患者均为KCNN4 c.1055G>A突变。目前基因型与表型间未见明确关联[3]。

本组21.7％患者继发铁过载，以肝脏重度沉积为主，其中8.7％累及心肌。PIEZO1通道参与铁代谢调控，功能获得性突变可能通过影响铁调素导致铁过载[21]。非裔人群中PIEZO1 p.Glu756del突变与铁过载相关[22]–[23]，但本队列未发现该变异。本研究未证实铁过载与性别、年龄、临床表型或基因型相关。铁过载患者肝功能异常发生率显著升高，且可进展为肝硬化，影响预后[24]。对于贫血不显著的DHS患者，静脉放血为去铁治疗首选，铁螯合剂亦有效。本组患者去铁治疗后贫血亦有改善。

与HS不同，脾切除术对DHS疗效有限，且可能增加DHS 1型患者血栓风险[25]。Stewart等[26]报道9例HST患者［其中5例为DHS，4例为过度水化（水肿）型遗传性口型红细胞增多症］脾切除后均出现血栓并发症，其中4例死亡。本队列中3例误诊为HS而接受脾切除/栓塞术的患者，术后溶血有所改善，但缓解程度不及典型HS患者，且未予抗凝治疗亦未发生血栓。鉴于中国人群血栓风险可能低于欧美，脾切除术在中国DHS患者中的风险与获益仍需进一步评估。

针对有症状的贫血患者，Gardos通道抑制剂（如西尼泊克）可能通过减少红细胞K^+^流失和脱水而成为潜在治疗选择[27]–[29]。

本研究存在单中心选择偏倚，轻症或典型病例可能未纳入，而铁过载等复杂病例比例较高。未来需开展全国多中心合作，以更全面揭示中国DHS的临床与遗传特征。

DHS易因贫血轻微、口型红细胞检出率低而误诊为HS，基因检测是确诊关键。脾切除术在中国DHS患者中未见明确血栓风险，但其适应证仍需谨慎评估。此外，DHS易合并肝铁过载，应定期监测并及时干预，以改善患者预后。
